# (Di­methyl­phosphor­yl)methanaminium hydrogen oxalate–oxalic acid (2/1)

**DOI:** 10.1107/S1600536814002931

**Published:** 2014-02-15

**Authors:** Sebastian Bialek, Rebecca Clemens, Guido J. Reiss

**Affiliations:** a Institut für Anorganische Chemie und Strukturchemie, Lehrstuhl II: Material- und Strukturforschung, Heinrich-Heine-Universität Düsseldorf, Universitätsstrasse 1, D-40225 Düsseldorf, Germany

## Abstract

The reaction of (di­methyl­phosphor­yl)methanamine (dpma) with oxalic acid in ethanol yielded the title solvated salt, C_3_H_11_NOP^+^·C_2_HO_4_^−^·0.5C_2_H_2_O_4_. Its asymmetric unit consists of one dpmaH^+^ cation, one hydrogen oxalate anion and a half-mol­ecule of oxalic acid located around a twofold rotation axis. The H atom of the hydrogen oxalate anion is statistically disordered over two positions that are *trans* to each other. The hydrogen oxalate monoanion is not planar (bend angle ∼16°) whereas the oxalic acid molecule shows a significantly smaller bend angle (∼7°). In the crystal, the components are connected by strong O—H⋯O and much weaker N—H⋯O hydrogen bonds, leading to the formation of layers extending parallel to (001). The structure was refined from a racemically twinned crystal with twin components in an approximate 1:1 ratio.

## Related literature   

For transition metal complexes of the cationic dpmaH^+^ ligand, see: Reiss (2013*a* ▶,*c* ▶). For simple dpmaH^+^ salts, see: Reiss & Jörgens (2012 ▶); Bianga *et al.* (2013 ▶); Buhl *et al.* (2013 ▶); Lambertz *et al.* (2013 ▶); Reiss (2013*b* ▶,*d* ▶).
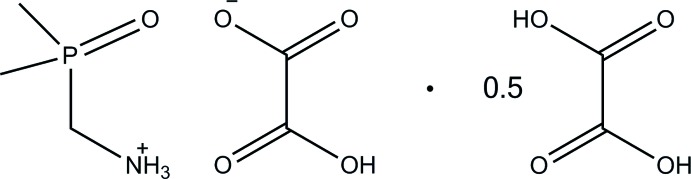


## Experimental   

### 

#### Crystal data  

C_3_H_11_NOP^+^·C_2_HO_4_^−^·0.5C_2_H_2_O_4_
*M* *_r_* = 242.14
Orthorhombic, 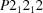

*a* = 11.1482 (6) Å
*b* = 13.0903 (7) Å
*c* = 7.0432 (4) Å
*V* = 1027.84 (10) Å^3^
*Z* = 4
Mo *K*α radiation
μ = 0.29 mm^−1^
*T* = 296 K
0.52 × 0.24 × 0.14 mm

#### Data collection  

Bruker APEXII CCD diffractometer
92924 measured reflections
3002 independent reflections
2990 reflections with *I* > 2σ(*I*)
*R*_int_ = 0.020

#### Refinement  

*R*[*F* ^2^ > 2σ(*F* ^2^)] = 0.019
*wR*(*F* ^2^) = 0.052
*S* = 1.05
3002 reflections
192 parameters
H atoms treated by a mixture of independent and constrained refinement
Δρ_max_ = 0.24 e Å^−3^
Δρ_min_ = −0.16 e Å^−3^
Absolute structure: refined as an inversion twin
Absolute structure parameter: 0.54 (7)

### 

Data collection: *APEX2* (Bruker, 2008 ▶); cell refinement: *SAINT* (Bruker, 2008 ▶); data reduction: *SAINT*; program(s) used to solve structure: *SHELXS97* (Sheldrick, 2008 ▶); program(s) used to refine structure: *SHELXL97* (Sheldrick, 2008 ▶); molecular graphics: *DIAMOND* (Brandenburg, 2012 ▶); software used to prepare material for publication: *publCIF* (Westrip, 2010 ▶).

## Supplementary Material

Crystal structure: contains datablock(s) I, New_Global_Publ_Block. DOI: 10.1107/S1600536814002931/wm5001sup1.cif

Structure factors: contains datablock(s) I. DOI: 10.1107/S1600536814002931/wm5001Isup2.hkl

Supporting information file. DOI: 10.1107/S1600536814002931/wm5001Isup3.cml

CCDC reference:

Additional supporting information:  crystallographic information; 3D view; checkCIF report

## Figures and Tables

**Table 1:** Hydrogen-bond geometry (Å, °)

*D*—H⋯*A*	*D*—H	H⋯*A*	*D*⋯*A*	*D*—H⋯*A*
O2—H2⋯O2^i^	0.77 (3)	1.73 (4)	2.4959 (17)	177 (4)
O4—H4⋯O4^ii^	0.81 (4)	1.67 (4)	2.4694 (18)	171 (4)
N1—H1*N*⋯O6^iii^	0.84 (2)	2.56 (2)	3.2106 (16)	135.4 (19)
N1—H1*N*⋯O7	0.84 (2)	2.27 (2)	2.9939 (17)	144 (2)
N1—H2*N*⋯O5	0.86 (2)	2.03 (2)	2.8760 (14)	168 (2)
N1—H3*N*⋯O3^iv^	0.91 (2)	1.92 (2)	2.8030 (15)	166.2 (19)
N1—H3*N*⋯O4^iv^	0.91 (2)	2.49 (2)	3.0419 (16)	120.0 (16)
O6—H6⋯O1^v^	0.89 (3)	1.59 (3)	2.4701 (14)	168 (3)

Symmetry codes: (i) 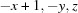; (ii) 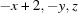; (iii) 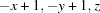; (iv) 
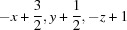
; (v) 
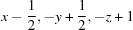
.

**Fig. 1.**
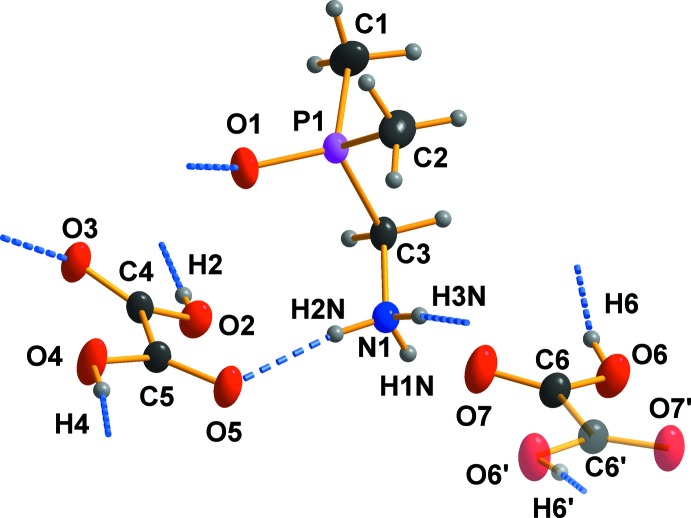
The molecular moieties of the title structure. Displacement ellipsoids are drawn at the 50% probability level. [Symmetry code: ' = 1 - x, 1 - y, z.]

**Fig. 2.**
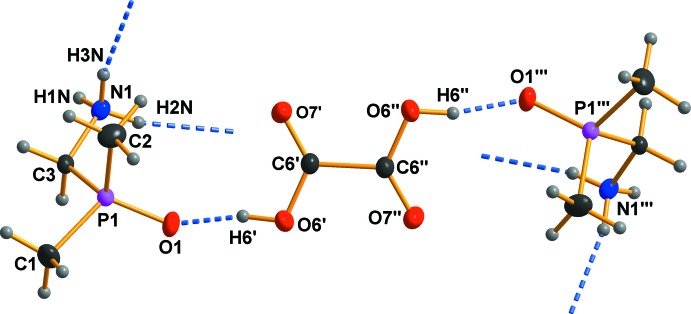
Two dpmaH+ cations connected via an oxalic acid molecule. Displacement ellipsoids as in Fig. 1. [Symmetry codes: ' = 0.5 + x, 0.5 - y, 1 - z; '' = 1.5 - x, -0.5 + y, 1 - z; ''' = 2 - x, -y, z.]

**Fig. 3.**
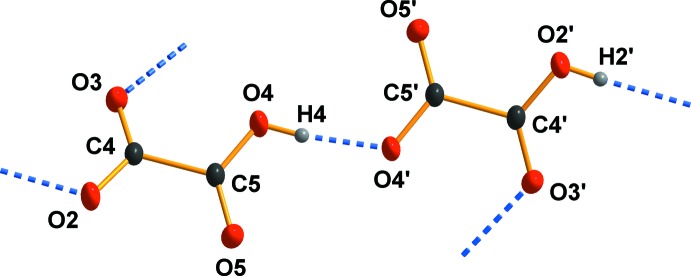
Part of the polar chain oriented along [100], built by head-to-tail connected hydrogen oxalate anions. For clarity, just one of the disordered hydrogen atoms is shown. Displacement ellipsoids as in Fig. 1. [Symmetry code: ' = 2 - x, -y, z.]

**Fig. 4.**
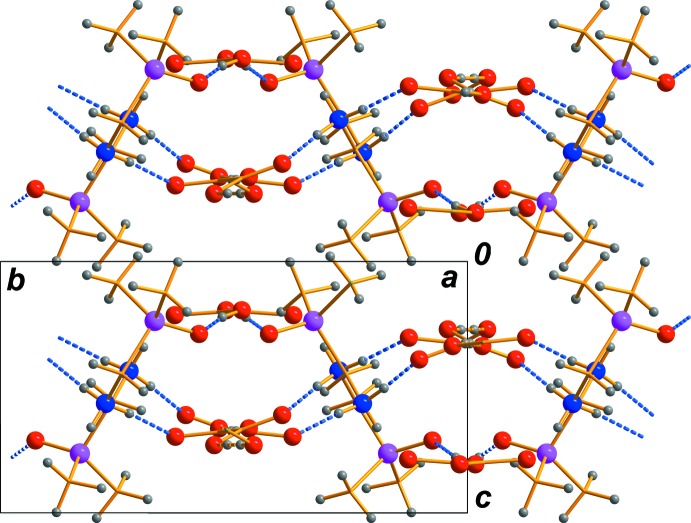
The two-dimensional framework parallel to (001) constructed by hydrogen bonding interactions (dashed lines).

## supplementary crystallographic information

### 1. Comment

The (dimethylphosphoryl)methanaminium (dpmaH^+^) cation is able to build various hydrogen-bonded, one dimensional structures (Bianga *et al.*, 2013; Buhl *et al.*, 2013; Lambertz *et al.*, 2013; Reiss, 2013*a*,*b*, Reiss & Jörgens, 2012). However, we have shown that structures with a higher dimensional cross-linking by hydrogen bonds are also possible (Reiss, 2013*c*,*d*). Furthermore, it has been shown that the dpmaH^+^ cation is able to coordinate transition metal cations by the oxygen atom of its phosphoryl group (Reiss, 2013*a*,*c*). In this contribution we present a further example of a dpmaH^+^ salt, 2(C_3_H_11_NPO^+^ C_2_HO_4_^-^)^.^C_2_H_2_O_4_, owing a complex hydrogen bonding scheme.

As illustrated in Fig. 1 the asymmetric unit of the title structure consists of one (dimethylphosphoryl)methanaminium (dpmaH^+^) cation, one hydrogen oxalate monoanion in general positions and one half of an oxalic acid molecule located around a twofold rotation axis. The geometric parameters of the dpmaH^+^ cation, the hydrogen oxalate monoanion and the oxalic acid molecule are generally in the expected ranges. The hydrogen oxalate monoanion is, as might be expected, not planar (bent angle ~ 16°). The oxalic acid molecule has an imposed twofold rotation symmetry. This neutral molecule shows significantly smaller bent angles (~7°) than the hydrogen oxalate monoanion. The neutral molecules, cations and anions are connected by strong O—H···O and much weaker N—H···O hydrogen bonds (Table 1). The O···O distances range from 2.4694 (18) Å to 2.4959 (17) Å whereas the N···O distances are 2.8030 (15) Å, 2.8760 (14) Å and 2.9939 (17) Å. The latter may be interpreted at least as a weak hydrogen bond.

For the further structural discussion we only consider the shorter hydrogen bonding connections (Fig. 1; O···O and N···O < 2.9 Å). The dpmaH^+^ cation appears, under this assumption, as a twofold hydrogen bond donor (connected to two anions) and a single hydrogen bond acceptor (connected to an oxalic acid molecule). As shown in Fig. 2, two dpmaH^+^ cations are connected to one oxalic acid molecule by two symmetry-related O—H···O hydrogen bonds of medium strength. The hydrogen oxalate monoanion acts as a single hydrogen bond donor and a twofold hydrogen bond acceptor. The hydrogen oxalate anions form head-to-tail connected polar chains running along [100] *via* medium strength O—H···O hydrogen bonds, as illustrated in Fig. 3. Caused by a 1:1 disorder of the hydrogen atom of this anion (attached to O2/O4) polar chains are present, which are oriented along and against [100], respectively. These chains are connected to the dpmaH^+^ cation through N—H···O hydrogen bonds into a two-dimensional-structure parallel to (001). Characteristic for this arrangement are gaps between the anion chains and the oxalic acid molecules and the hydrophobic areas where neighbouring layers are facing each other (Fig. 4).

### 2. Experimental

The title compound, 2(C_3_H_11_NPO^+^ C_2_HO_4_^-^)^.^C_2_H_2_O_4_, was prepared by dissolving 1.01 g dpma and 1.17 g oxalic acid in 5 ml ethanol. Within a few days under ambient conditions, colourless crystals were obtained by slow evaporation of the solvent.

### 3. Refinement

For the disordered hydrogen atom at the hydrogen oxalate anion the split positions were refined freely with a ratio of 1:1. One common *U*_iso_ value was refined for both sites. All other H atoms were identified in difference syntheses and refined freely with individual *U*_iso_ values.

### Crystal data

**Table:**

C_3_H_11_NOP^+^·C_2_HO_4_^−^·0.5C_2_H_2_O_4_	*D*_x_ = 1.565 Mg m^−^^3^
*M**_r_* = 242.14	Mo *K*α radiation, λ = 0.71073 Å
Orthorhombic, *P*2_1_2_1_2	Cell parameters from 9422 reflections
*a* = 11.1482 (6) Å	θ = 2.9–35.9°
*b* = 13.0903 (7) Å	µ = 0.29 mm^−^^1^
*c* = 7.0432 (4) Å	*T* = 296 K
*V* = 1027.84 (10) Å^3^	Lath, colourless
*Z* = 4	0.52 × 0.24 × 0.14 mm
*F*(000) = 508	

### Data collection

**Table:**

Bruker APEXII CCD diffractometer	2990 reflections with *I* > 2σ(*I*)
Radiation source: sealed tube	*R*_int_ = 0.020
Graphite monochromator	θ_max_ = 30.0°, θ_min_ = 2.4°
φ and ω scans	*h* = −15→15
92924 measured reflections	*k* = −18→18
3002 independent reflections	*l* = −9→9

### Refinement

**Table:**

Refinement on *F*^2^	Secondary atom site location: difference Fourier map
Least-squares matrix: full	Hydrogen site location: difference Fourier map
*R*[*F*^2^ > 2σ(*F*^2^)] = 0.019	H atoms treated by a mixture of independent and constrained refinement
*wR*(*F*^2^) = 0.052	*w* = 1/[σ^2^(*F*_o_^2^) + (0.030*P*)^2^ + 0.150*P*] where *P* = (*F*_o_^2^ + 2*F*_c_^2^)/3
*S* = 1.05	(Δ/σ)_max_ = 0.001
3002 reflections	Δρ_max_ = 0.24 e Å^−^^3^
192 parameters	Δρ_min_ = −0.16 e Å^−^^3^
0 restraints	Absolute structure: refined as an inversion twin
Primary atom site location: structure-invariant direct methods	Absolute structure parameter: 0.54 (7)

### Special details

**Table:**

Geometry. All e.s.d.'s (except the e.s.d. in the dihedral angle between two l.s. planes) are estimated using the full covariance matrix. The cell e.s.d.'s are taken into account individually in the estimation of e.s.d.'s in distances, angles and torsion angles; correlations between e.s.d.'s in cell parameters are only used when they are defined by crystal symmetry. An approximate (isotropic) treatment of cell e.s.d.'s is used for estimating e.s.d.'s involving l.s. planes.
Refinement. Refined as a 2-component inversion twin.

### Fractional atomic coordinates and isotropic or equivalent isotropic displacement parameters (Å^2^)

**Table:**

	*x*	*y*	*z*	*U*_iso_*/*U*_eq_	Occ. (<1)
P1	0.59355 (2)	0.16995 (2)	0.76357 (4)	0.02120 (7)	
O1	0.66126 (9)	0.07493 (7)	0.71183 (16)	0.0320 (2)	
C1	0.45282 (14)	0.14728 (13)	0.8751 (3)	0.0401 (3)	
H1A	0.402 (3)	0.101 (2)	0.800 (4)	0.077 (8)*	
H1B	0.415 (2)	0.216 (2)	0.902 (4)	0.066 (7)*	
H1C	0.467 (3)	0.120 (2)	0.997 (4)	0.069 (8)*	
C2	0.67941 (16)	0.25082 (12)	0.9144 (2)	0.0362 (3)	
H2A	0.696 (3)	0.208 (2)	1.021 (4)	0.066 (8)*	
H2B	0.637 (2)	0.3147 (18)	0.947 (3)	0.048 (6)*	
H2C	0.754 (2)	0.268 (2)	0.851 (4)	0.069 (8)*	
C3	0.55056 (10)	0.24000 (9)	0.55333 (18)	0.0226 (2)	
H3A	0.5027 (19)	0.1946 (16)	0.482 (3)	0.039 (5)*	
H3B	0.5075 (19)	0.3008 (15)	0.593 (3)	0.038 (5)*	
N1	0.65260 (10)	0.27530 (8)	0.43508 (17)	0.0253 (2)	
H1N	0.6245 (19)	0.3065 (17)	0.341 (3)	0.043 (5)*	
H2N	0.694 (2)	0.2243 (17)	0.397 (3)	0.040 (5)*	
H3N	0.6989 (19)	0.3213 (16)	0.497 (3)	0.037 (5)*	
O2	0.60038 (7)	0.04219 (7)	0.26969 (15)	0.02816 (18)	
O3	0.67262 (8)	−0.10318 (7)	0.38932 (17)	0.0314 (2)	
O4	0.89578 (8)	−0.03192 (7)	0.33626 (19)	0.0354 (2)	
O5	0.82187 (8)	0.12549 (7)	0.30304 (17)	0.0312 (2)	
H2	0.539 (3)	0.014 (3)	0.272 (5)	0.035 (7)*	0.5
H4	0.962 (3)	−0.007 (3)	0.328 (5)	0.035 (7)*	0.5
C4	0.68433 (9)	−0.01553 (8)	0.33008 (17)	0.0212 (2)	
C5	0.81095 (9)	0.03311 (9)	0.32141 (17)	0.0212 (2)	
H6	0.283 (2)	0.476 (2)	0.220 (4)	0.070 (8)*	
O6	0.34552 (9)	0.51669 (8)	0.19359 (19)	0.0380 (3)	
O7	0.45436 (10)	0.37300 (8)	0.2131 (2)	0.0404 (3)	
C6	0.44455 (11)	0.46465 (10)	0.20114 (19)	0.0271 (2)	

### Atomic displacement parameters (Å^2^)

**Table:**

	*U*^11^	*U*^22^	*U*^33^	*U*^12^	*U*^13^	*U*^23^
P1	0.01823 (12)	0.01867 (12)	0.02670 (13)	0.00132 (9)	0.00031 (11)	−0.00042 (10)
O1	0.0279 (4)	0.0260 (4)	0.0421 (5)	0.0105 (3)	−0.0041 (4)	−0.0037 (4)
C1	0.0283 (6)	0.0398 (8)	0.0521 (9)	0.0016 (6)	0.0134 (7)	0.0109 (7)
C2	0.0451 (8)	0.0336 (7)	0.0299 (6)	−0.0056 (6)	−0.0105 (6)	−0.0022 (5)
C3	0.0179 (5)	0.0198 (5)	0.0300 (5)	0.0019 (4)	−0.0031 (4)	−0.0012 (4)
N1	0.0271 (5)	0.0211 (4)	0.0275 (5)	0.0033 (4)	0.0014 (4)	0.0007 (4)
O2	0.0134 (3)	0.0272 (4)	0.0439 (5)	−0.0003 (3)	−0.0022 (4)	0.0045 (4)
O3	0.0184 (4)	0.0201 (4)	0.0557 (6)	−0.0023 (3)	0.0030 (4)	0.0049 (4)
O4	0.0126 (3)	0.0220 (4)	0.0715 (7)	0.0001 (3)	−0.0009 (4)	0.0047 (4)
O5	0.0194 (4)	0.0187 (4)	0.0556 (6)	−0.0024 (3)	0.0005 (4)	0.0019 (4)
C4	0.0131 (4)	0.0197 (5)	0.0307 (5)	−0.0016 (4)	0.0017 (4)	−0.0027 (4)
C5	0.0129 (4)	0.0203 (4)	0.0305 (5)	−0.0011 (4)	0.0010 (4)	0.0004 (4)
O6	0.0199 (4)	0.0279 (5)	0.0661 (8)	−0.0022 (3)	0.0020 (4)	0.0074 (5)
O7	0.0291 (5)	0.0237 (4)	0.0685 (8)	−0.0027 (4)	−0.0058 (5)	0.0032 (5)
C6	0.0224 (5)	0.0237 (5)	0.0351 (6)	−0.0033 (4)	−0.0017 (4)	0.0014 (4)

### Geometric parameters (Å, º)

**Table:**

P1—O1	1.5000 (9)	N1—H2N	0.86 (2)
P1—C2	1.7791 (15)	N1—H3N	0.91 (2)
P1—C1	1.7796 (15)	O2—C4	1.2759 (14)
P1—C3	1.8064 (12)	O2—H2	0.77 (3)
C1—H1A	0.99 (3)	O3—C4	1.2278 (15)
C1—H1B	1.01 (3)	O4—C5	1.2767 (14)
C1—H1C	0.95 (3)	O4—H4	0.81 (4)
C2—H2A	0.96 (3)	O5—C5	1.2223 (14)
C2—H2B	0.99 (2)	C4—C5	1.5497 (15)
C2—H2C	0.97 (3)	O6—C6	1.2984 (15)
C3—N1	1.4836 (16)	O6—H6	0.89 (3)
C3—H3A	0.94 (2)	O7—C6	1.2076 (15)
C3—H3B	0.97 (2)	C6—C6^i^	1.544 (2)
N1—H1N	0.84 (2)		
			
O1—P1—C2	111.58 (7)	N1—C3—H3B	106.5 (12)
O1—P1—C1	114.37 (7)	P1—C3—H3B	108.3 (12)
C2—P1—C1	108.05 (9)	H3A—C3—H3B	112.9 (17)
O1—P1—C3	110.81 (6)	C3—N1—H1N	107.9 (15)
C2—P1—C3	109.28 (7)	C3—N1—H2N	110.4 (15)
C1—P1—C3	102.28 (7)	H1N—N1—H2N	109 (2)
P1—C1—H1A	112.1 (17)	C3—N1—H3N	112.0 (13)
P1—C1—H1B	107.9 (15)	H1N—N1—H3N	105.7 (19)
H1A—C1—H1B	114 (2)	H2N—N1—H3N	111.2 (19)
P1—C1—H1C	108.4 (18)	C4—O2—H2	111 (3)
H1A—C1—H1C	110 (2)	C5—O4—H4	113 (3)
H1B—C1—H1C	104 (2)	O3—C4—O2	126.07 (10)
P1—C2—H2A	103.0 (16)	O3—C4—C5	119.62 (10)
P1—C2—H2B	112.4 (13)	O2—C4—C5	114.31 (10)
H2A—C2—H2B	114 (2)	O5—C5—O4	126.48 (10)
P1—C2—H2C	109.1 (17)	O5—C5—C4	120.09 (10)
H2A—C2—H2C	110 (2)	O4—C5—C4	113.42 (9)
H2B—C2—H2C	109 (2)	C6—O6—H6	110.0 (18)
N1—C3—P1	114.51 (8)	O7—C6—O6	126.95 (12)
N1—C3—H3A	109.3 (12)	O7—C6—C6^i^	121.53 (15)
P1—C3—H3A	105.6 (12)	O6—C6—C6^i^	111.48 (13)
			
O1—P1—C3—N1	−61.08 (10)	O2—C4—C5—O5	16.86 (17)
C2—P1—C3—N1	62.27 (11)	O3—C4—C5—O4	15.53 (18)
C1—P1—C3—N1	176.61 (10)	O2—C4—C5—O4	−163.53 (12)
O3—C4—C5—O5	−164.08 (13)		

Symmetry code: (i) −*x*+1, −*y*+1, *z*.

### Hydrogen-bond geometry (Å, º)

**Table:**

*D*—H···*A*	*D*—H	H···*A*	*D*···*A*	*D*—H···*A*
O2—H2···O2^ii^	0.77 (3)	1.73 (4)	2.4959 (17)	177 (4)
O4—H4···O4^iii^	0.81 (4)	1.67 (4)	2.4694 (18)	171 (4)
N1—H1*N*···O6^i^	0.84 (2)	2.56 (2)	3.2106 (16)	135.4 (19)
N1—H1*N*···O7	0.84 (2)	2.27 (2)	2.9939 (17)	144 (2)
N1—H2*N*···O5	0.86 (2)	2.03 (2)	2.8760 (14)	168 (2)
N1—H3*N*···O3^iv^	0.91 (2)	1.92 (2)	2.8030 (15)	166.2 (19)
N1—H3*N*···O4^iv^	0.91 (2)	2.49 (2)	3.0419 (16)	120.0 (16)
O6—H6···O1^v^	0.89 (3)	1.59 (3)	2.4701 (14)	168 (3)

Symmetry codes: (i) −*x*+1, −*y*+1, *z*; (ii) −*x*+1, −*y*, *z*; (iii) −*x*+2, −*y*, *z*; (iv) −*x*+3/2, *y*+1/2, −*z*+1; (v) *x*−1/2, −*y*+1/2, −*z*+1.
